# FINANCIAL HARDSHIP IN OLDER CHINESE CANCER SURVIVORS

**DOI:** 10.1093/geroni/igz038.3137

**Published:** 2019-11-08

**Authors:** Mingzhu Su, Nengliang Yao, Xiaojie Sun

**Affiliations:** Shandong University, Jinan, Shandong, China

## Abstract

Purpose: To estimate the proportion of Chinese cancer survivors experienced financial hardship and then examine whether older age was associated with financial hardship. Methods: We surveyed 965 cancer survivors 30 to 64 years of age and 643 cancer survivors age >=65 years in China. Cancer survivors were asked whether (1) they have borrowed money because of cancer, its treatment, and lasting effects of treatment and (2) they have forgone some cancer-related medical care because of cost. Multi-variable logistic regression models were used to examine factors associated with financial hardship. Results: About 44% of cancer survivors older than 65 borrowed money because of cancer, and 18% had borrowed more than 20,000 CNY (about 2,900USD, the disposable personal income in China in 2015 was about 22,000 CNY). In contrast, 54% of younger patients (P<0.01) had cancer-related debts, and 32% had to borrow more than 20,000 CNY. About 11% of cancer survivors have forgone cancer care in both age groups. The logistic regression analyses show that being 65 or older was 43% less likely to report cancer-related debts than younger patients (OR=0.57, 95% CI: 0.44-0.73). Among older cancer survivors, those who were older than 75, female, and had Urban Employee Medical Insurance and higher family income were less likely to report financial hardship. Conclusion: Older cancer survivors in China experience significant financial hardship, but not as striking as younger patients. Additional research is needed to analyze whether the finding is associated with the Chinese family structure and traditional filial piety culture.

